# About the International Asteroid Warning Network (IAWN) and the Space Mission Planning Advisory Group (SMPAG)

**DOI:** 10.1038/s41467-024-48600-x

**Published:** 2024-06-06

**Authors:** Detlef V. Koschny, Kelly E. Fast, Romana Kofler

**Affiliations:** 1 TU Munich, Lunar and Planetary Exploration, Lise-Meitner-Str. 9, 85521 Ottobrunn, Germany; 2 Planetary Defense Coordination Office/Planetary Science Division, NASA Headquarters, 300 E Street SW, Washington, DC 20546 USA; 3 United Nations Office for Outer Space Affairs (UNOOSA), United Nations Office at Vienna, Vienna International Centre, P.O. Box 500, A-1400 Vienna, Austria

**Keywords:** Policy, Asteroids, comets and Kuiper belt

## Abstract

Atmospheric entry of asteroids or comets can cause significant damage to Earth. Two international bodies, the International Asteroid Warning Network (IAWN), and the Space Mission Planning Advisory Group (SMPAG) are working on dealing with potential threats.

## Planetary defence

Besides planets and dwarf planets, a large number of smaller objects known as asteroids (typically rocky or metallic objects) and comets (typically icy objects) exist in our solar system. The Minor Planet Center, endorsed by the International Astronomical Union, holds position measurements for about 1.5 million such objects^1^. The Minor Planet Center currently has classified more than 34,600 as near-Earth objects, with orbits that bring them closer than 1.3 astronomical units (one astronomical unit is the distance between Earth and the Sun) to the Sun and thus possibly close to our planet. While they are scientifically very useful to understand the formation and evolution of our solar system, near-Earth objects (NEOs) might also pose a threat when impacting our home planet. Luckily, we are currently not aware of any potentially impacting object that could cause significant damage.

The minimum size of an NEO that could do damage should it impact is around 10 m. Such objects impact Earth typically every few years to tens of years. Larger objects generate more damage but are rarer. For instance, in February 2013, an approximately 20 m-sized asteroid broke up as it impacted Earth’s atmosphere above the city of Chelyabinsk. The shock wave caused damage, and about 1500 people were injured^2^. Events like this happen on average every 10 to 100 years somewhere on our planet. The impact of an object around 40 m in size could result in city-wide damage. Such impacts occur every 100 to 1000 years^3^. Only about 1% of the objects in this size range are known, therefore, such an impact could happen any time.

The activity to deal with this topic is called ‘Planetary Defence’. In 2013, two groups were recommended by the United Nations to address this topic: The International Asteroid Warning Network (IAWN), and the Space Mission Planning Advisory Group (SMPAG). This was a result of a thorough discussion process, which started in the year 2000, following the UNISPACE III conference. The recommendations for an international response to the near-Earth object impact threat were endorsed by the Committee on the Peaceful Uses of Outer Space (COPUOS) in June 2013 and by the United Nations General Assembly in December 2013. For a detailed description of the process to establish these groups, see Kofler et al.^4^.

## What are the main tasks of IAWN and SMPAG?

IAWN^5^ is a network of organizations or individuals that are active in searching for and tracking near-Earth objects, computing and predicting their orbits, computing and predicting close Earth flybys or even impacts, and computing impact effects from their observed properties. IAWN is also responsible for generating warning messages in case of an asteroid impact threat, and coordination with emergency response agencies. SMPAG^6^ consists of a group of delegations from space-faring nations. Their task is “… to prepare for an international response to a NEO impact threat through the exchange of information, development of options for collaborative research and mission opportunities, and NEO threat mitigation planning activities”^7^.

The United Nations Office of Outer Space Affairs (UNOOSA)^8^ also plays a major role in the process. It acts as a secretariat for SMPAG. It is also represented at all IAWN meetings and is closely involved in the information flow.

IAWN’s main task is to warn of a possible impact threat, if the following criteria are reached: an impact probability of >1% within the next 20 years, for an object larger than about 10 meters in size, IAWN would produce alerts with summary information for SMPAG and for UNOOSA, which will be distributed via UNOOSA’s diplomatic channels to all UN member countries across our planet. These alerts would also be available via the IAWN webpage.

**Fig. 1:**
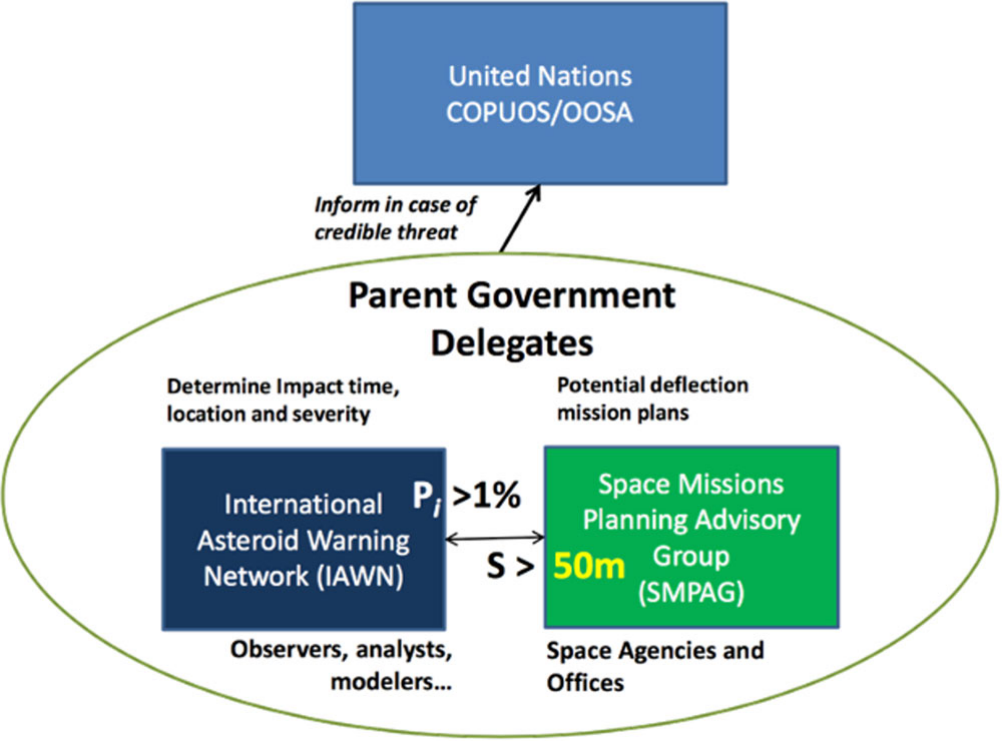
Conceptual diagramme of the existing UN-endorsed groups dealing with a possible asteroid impact threat. *P*_*i*_ denotes the probability of an impact; *S* the size. IAWN is a network of organizations that observe asteroids, compute their orbits, and predict their positions in the future. They compute possible impact effects and produce alerts and warnings. SMPAG becomes active for larger objects to plan and propose a possible space-based response.

In case the object is large enough to justify space-based activities, SMPAG becomes active. The criteria for SMPAG are an impact probability of >1%, within the next 50 years, for objects larger than ~50 m. For smaller objects, it is assumed that it will be more efficient to prepare for the threat via ground-based response (see Fig. 1).

## What happens when an asteroid threat is found?

Let’s assume, as an example, that there is an asteroid out there with a size of about 100 m and a 2% chance of hitting our planet 20 years from now. It would likely be discovered by one of the major asteroid surveys and reported to the Minor Planet Center^1^. From there, follow-up observers worldwide would provide further observations of the asteroid’s position to the Minor Planet Center, and within a short time span (typically after a few days) the orbit would be known to a reasonable accuracy – allowing the estimate of impact with the assumed probability of 2%. These estimates are currently done mainly by the Center for NEO Studies at the Jet Propulsion Laboratory^9^ of the National Aeronautics and Space Administration (NASA) and the NEO Coordination Centre^10^ of the European Space Agency (ESA). Using the size estimated from the brightness of the asteroid, different international teams would start computing possible impact effects.

Once the teams are sure that the impact prediction is reliable – which depends on the orbital characteristics of the object and the amount and quality of the observational data – IAWN would prepare an alert message coordinated with the members of the IAWN Steering Committee of about 10 members. Once agreed, the message would be passed to the chair of SMPAG and to UNOOSA, which would distribute it via a note verbale to all member states. In parallel, space agencies and other participants in IAWN would inform locally according to their own national policies. For example, NASA would inform the United States government and federal agencies such as the Federal Emergency Management Agency (FEMA). ESA would inform their member delegations and their emergency response agencies. Since the data at the Minor Planet Center and the ESA and NASA data centres are public, it is more than likely that such a scenario would be in the news and on social media before these official notifications could be made. However, IAWN’s goal is to have the most accurate and current information from the worldwide observing and modeling community to convey to the international community through UNOOSA. Although detailed procedures are still to be worked out, a large part of the alert production is automated, and accurate notifications could be coordinated and distributed in a matter of hours or less, as needed.

In this example case, SMPAG would also be activated given the size and timing of the predicted impact. The SMPAG chair would call for a meeting to discuss the issue. The members would, according to their expertise, prepare possible scenarios for reconnaissance and deflection missions. In coordinated discussions, a recommendation would be produced on how to best address the threat. This recommendation would be given to decision-makers – leaders of space agencies, national delegations, etc. Preparing a first recommendation is expected to take weeks to months given the details involved in designing a mission concept, but it could be shorter if time were pressing.

In parallel, IAWN would encourage astronomers to collect more data and support a coordinated analysis of the results. In case of major changes in the findings, new information would be disseminated as with the initial notification.

The information from IAWN and the recommendations by SMPAG can be utilized by decision-makers in governments as they plan their response to the threat, ideally in an internationally collaborative manner.

## Current activities and outlook

Even though no serious impact threat is currently known, the worldwide community works to be prepared. Members of IAWN search for and observe the objects, identify those which come close to the Earth and estimate impact effects. IAWN will produce alerts and warnings if needed. SMPAG brings together the expertise of the space-faring nations and it will study and propose a possible space-based response to an impact threat. This could be a reconnaissance mission to better characterize the object, and/or deflection missions to avoid a possible impact altogether.

Both groups regularly meet twice per year. Typically, one meeting in the year takes place in Vienna, Austria, on the margins of the meetings of the Scientific and Technical Subcommittee of the United Nations Committee on the Peaceful Uses of Outer Space. The group’s web pages provide reports on these meetings shortly after they took place.

IAWN regularly performs exercises with their network members, such as observing the physical properties of a near-Earth asteroid or checking the quality of position measurements by observing a well-known asteroid^11–15^. For example, providing proper time stamps with astrometric (positional) observations can be critical in the accurate determination of asteroid orbits, in particular for objects close to the Earth. Observers were invited to perform astrometric observations of asteroids as well as Global Navigation System satellites during two campaigns to assess the timing errors of participating observatories and the overall accuracy of the global network.

Exercises that involve an asteroid impact scenario have taken place at international conferences like the Planetary Defense Conference, and within national governments. Both ESA and NASA have performed exercises with their national contacts, e.g. NASA with FEMA^16^, or ESA with the German Space Situational Awareness Centre. The latter resulted in the development of an automated alert interface, using computer-readable files in addition to a human-readable alert sheet called ‘Close Approaches Fact Sheet’^17^.

A first international exercise with decision-makers (e.g. agency directors) was performed at the Planetary Defense Conference 2023^18,19^. A simulated impact threat scenario was presented to the decision-makers. IAWN presented its warning, and SMPAG presented the result of a presumed discussion after being notified. SMPAG recommended a deflection option for the scenario based on a nuclear explosive device, which it found to have the highest probability of success. However, SMPAG also suggested further study of conventional deflection options. These were then discussed by the decision-makers. IAWN and SMPAG will continue to expand the participants of their exercises to real decision-makers (e.g., space agency leadership or politicians).

SMPAG is currently performing an exercise to identify detailed response procedures within member agencies or offices and encourages the exercise participants to interact with higher-level internal decision-makers and involve them as much as possible. A report of the first part of the exercise is under preparation and will be posted on the SMPAG website^5^.

Various mission concepts would be possible for deflecting an asteroid, such as in the previously mentioned threat scenario during the Planetary Defense Conference. To name just a few: A nuclear explosive device would be a nuclear detonation close to the asteroid, however, with difficult political and legal implications. An ion-beam shepherd is a technique where a beam from an ion engine would slowly push the asteroid. However, its feasibility is not yet demonstrated. Another method is a kinetic impactor where a heavy, high-speed spacecraft would impact the asteroid. The momentum transfer would alter the object’s trajectory. NASA’s DART mission (Double-Asteroid Redirection Test) impacted the smaller object of a double asteroid to test this deflection method in the year 2022^20^. The upcoming ‘Hera’ mission of ESA^21^ will provide the necessary physical property information of the asteroid system to fully understand the effect of the test. These missions (and future deflection test missions) provide important information, in particular for SMPAG to consider for recommending deflection methods.

An additional opportunity to study larger near-Earth asteroids is the upcoming close flyby of asteroid Apophis (about 370 m in size) in April 2029. It will fly by at a distance of around 32000 km to Earth’s surface, smaller than the distance to the belt of geostationary satellites. Studying Apophis from ground and space assets could yield valuable information about its physical properties, including structural information from possible changes on its surface due to stresses from its orbit-altering swing by Earth. The spacecraft from the NASA mission OSIRIS-REx, renamed to OSIRIS-APEX, will rendezvous with Apophis after its closest approach. Discussions for additional missions, in particular missions that would arrive before the close flyby, are currently ongoing in the community and with space agencies. IAWN is planning a worldwide campaign to observe and study Apophis during its close approach. It conducted a similar campaign during a distant approach to Earth in 2021 when radar observations and orbit analysis determined that Apophis poses no impact threat to Earth for at least the next century.

At the time of this writing, there is no known object larger than 10 m that poses a serious impact threat to our planet in the next century^22^. However, in the unlikely event that a large asteroid is discovered that poses a significant impact threat, the international collaborations of IAWN and SMPAG will share information on the threat and recommend potential space mission responses. IAWN and SMPAG will continue to look for opportunities to inform decision-makers (e.g., through asteroid impact exercises) so that an asteroid impact threat will not simply be perceived as the subject of movies, but a real-world hazard with real-world response possibilities.
